# Direct synthesis of amides and imines by dehydrogenative homo or cross-coupling of amines and alcohols catalyzed by Cu-MOF†

**DOI:** 10.1039/d1ra03142b

**Published:** 2021-06-10

**Authors:** Soheil Zamani Anbardan, Javad Mokhtari, Ahmad Yari, Abolfazl Hassani Bozcheloei

**Affiliations:** Department of Chemistry, Science and Research Branch, Islamic Azad University P. O. Box 14515/775 Tehran Iran j.mokhtari@srbiau.ac.ir

## Abstract

Oxidative dehydrogenative homo-coupling of amines to imines and cross-coupling of amines with alcohols to amides was achieved with high to moderate yields at room temperature in THF using Cu-MOF as an efficient and recyclable heterogeneous catalyst under mild conditions. Different primary benzyl amines and alcohols could be utilized for the synthesis of a wide variety of amides and imines. The Cu-MOF catalyst could be recycled and reused four times without loss of catalytic activity.

## Abstract 2

Oxidative dehydrogenative homo or cross-coupling of amines with alcohols to imines and amides was achieved with high to moderate yields at room temperature using Cu-MOF as an efficient and recyclable heterogeneous catalyst.

## Graphical abstract

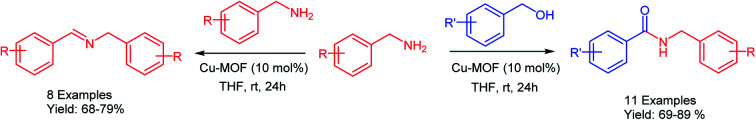

## Introduction

Imines are of great importance for the synthesis of chemical and biologically active compounds such as amines, chiral amines, amides, pyrrolines, hydroxyamines, and oxaziridines.^1–3^ Typically for the synthesis of imines, a mixture of an aldehyde or ketone with an amine in the presence of a catalyst is required. On the other hand, many useful oxidation methods for the synthesis of imines have been developed^4^ which include dimerization of primary amines^5–10^ oxidation of secondary amines^11–14^ and other methodologies.^15–23^

**Scheme 1:**
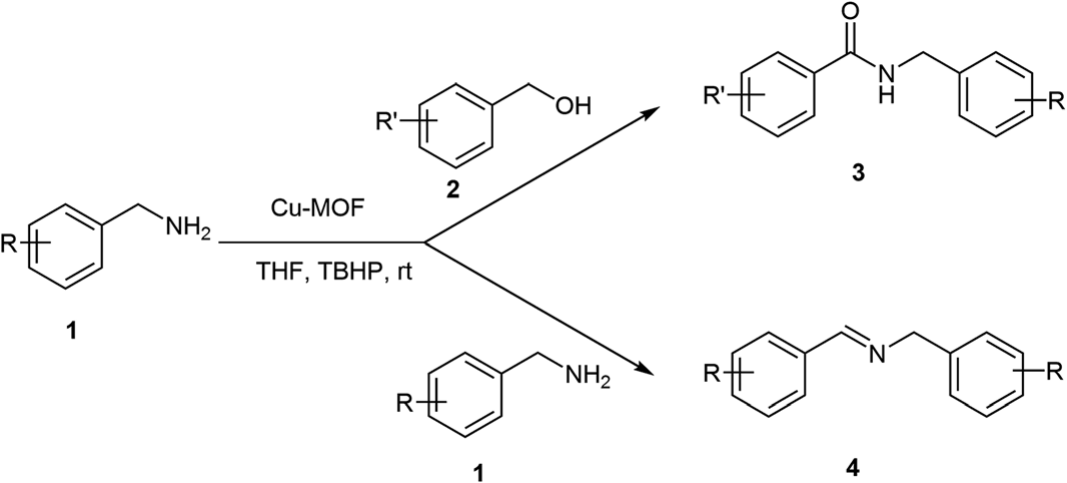
Oxidative homo and cross-coupling of amine with alcohols catalyzed by Cu-MOF.

The formation of amide bonds is one of the most commonly used organic reactions due to the widespread presence of this functional group in natural products, pharmaceutical compounds, and synthetic polymers.^24^ Usually, amides are synthesized by the reaction of an amine and carboxylic acid, which needs a coupling reagent^25^ or conversion into reactive derivatives.^26^ These methods have several drawbacks, such as the use of hazardous and expensive reagents, generating stoichiometric amounts of waste which lead to environmental problems. To solve these problems, there is great interest in the development of atom economic and environmentally friendly routes for the synthesis of amides such as named reactions like the Beckmann rearrangement,^27^ the Schmidt reaction^28^ and Ugi^29^ reaction. A more recent method has considered the use of direct oxidative amidation from alcohols and aldehydes.^30–41^ However, these methods require the use of expensive transition metals as catalyst and in some cases the reactions require hazardous, expensive, dual catalyst and in most cases is a problem and the catalyst is not recyclable.^42^ Therefore, the search of a green, low cost and heterogeneous catalyst system remains a challenge. Recently, MOFs have attracted much more attention due to their structural and chemical diversities and have become very popular in the diverse research areas such as catalysis,^43^ drug delivery^44^ gas adsorption and storage^45^ and *etc.* As part of our ongoing work to develop catalytic activity of metal–organic frameworks (MOFs) as efficient heterogeneous, green and recyclable catalyst in organic synthesis^46^ we report herein an improved oxidative homo-coupling of amines to imines and oxidative cross-coupling of amine with alcohols to amides, using inexpensive and readily available Cu_2_(BDC)_2_(DABCO) as the recyclable catalysts and TBHP as oxidant (Scheme 1).

## Experimental section

### Materials and methods

All the chemicals were purchased from commercial sources and used without further purification. Cu_2_(BDC)_2_(DABCO) was synthesized according to the our previously reported procedure.^46*a*^ All reactions were monitored by thin layer chromatography (TLC) using plates coated with Merck 60 HF254 silica under UV light. Melting points were measured on an Electrothermal 9100 apparatus. ^1^H-NMR spectra were recorded with BRUKER DRX 400 and 500-AVANCE FT-NMR instrument (CDCl_3_ solution) at 400 MHz and 500 MHz, respectively. Scanning electron microscope (SEM) images were captured with a ZEISS scanning electron microscope at 30 kV with gold coating. X-ray powder diffraction (XRD) measurements were performed using an X'pert MPD. Philips diffractometer with Cu radiation source (*λ* = 1.54050 Å) at 40 kV voltage and 40 mA current.

### Synthesis of amides *via* dehydrogenative cross-coupling of amines and alcohols catalyzed by Cu_2_(BDC)_2_(DABCO)

To a solution of amines (1 mmol) and alcohols (1 mmol) in THF (5 ml) was added Cu_2_(BDC)_2_DABCO (10% mol) and TBHP 70% (2 mmol) and the reaction mixture was stirred at room temperature for 24 h. The reaction progress was monitored by TLC. After the reaction was completed, the catalyst was filtered and the filtrate was evaporated under reduced pressure, and the residue was purified using silicagel column chromatography (hexane/ethyl acetate (3 : 1)).

### Selected spectral data

*N*-Benzylbenzamide (3a), white solid; yield: 78%; ^1^H NMR (500 MHz, CDCl_3_): *δ* 7.81 (d, *J* = 7.1 Hz, 1H, CH of Ar), 7.43–7.45 (m, 3H, CH of Ar), 7.36–7.37 (m, 4H, CH of Ar), 7.29 (d, *J* = 7.1 Hz, 2H), 6.45 (s, 1H, NH), 4.85 (s, 2H, benzylic CH_2_).

### Synthesis of imines *via* dehydrogenative homo-coupling of amines catalyzed by Cu_2_(BDC)_2_(DABCO)

To a solution of amines (2 mmol) in THF (5 ml) was added Cu_2_(BDC)_2_DABCO (10% mol) and TBHP 70% (4 mmol) and the reaction temperature was stirred at room temperature for 24 h. The reaction progress was monitored by TLC. After reaction completion, catalyst filtered and filtrate was evaporated under reduced pressure, and the residue was purified using silicagel column chromatography (hexane/ethyl acetate (3 : 1)).

*N*-(Benzylidene)benzylamine (4a).^47^^1^H NMR (400 MHz, CDCl_3_): *δ* 8.44 (s, 1H, CH), 7.97–7.76 (m, 2H, CH of Ar), 7.57–7.42 (m, 3H, CH of Ar), 7.40 (d, *J* = 4.4 Hz, 4H, CH of Ar), 7.34–7.27 (m, 1H, CH of Ar), 4.88 (s, 2H, benzylic CH_2_).

## Result and discussion

Cu_2_(BDC)_2_DABCO (Cu-MOF) catalysts were prepared and characterized according to our previous work.^46*a*^ The synthetic Cu_2_(BDC)_2_DABCO was employed as a catalyst in the dehydrogenative homo-coupling of amines to imines and cross-coupling of amines with alcohols to amides. At the first, for getting to the optimum reaction conditions, various parameters such as solvent, amount of the catalyst and oxidant were examined for the model reactions (Table 1). Three solvent including THF, DMF and CH_3_CN were investigated for this homo-coupling reaction in presence of Cu-MOF as catalyst and TBHP as oxidant. The results are summarized in the Table 1 and can be seen that the maximum yield was obtained in the THF as solvent (Table 1, entries 4–6). For the study of the amount of catalyst in this reaction, 5, 10 and 20 mol% of the catalyst was used and as shown in Table 1, 10 mol% of Cu-MOF was the best for the synthesis of imines and amides (Table 1, entries 5, 7) and the higher amounts of the catalyst did not significantly effect on the reaction yield. TBHP was used as oxidant because of our previous experience in oxidative coupling reactions.^43^ Control experiments revealed that in the absence of Cu-MOF as catalyst (Table 1, entry 10) and TBHP as oxidant (Table 1, entry 11) the product 3a and 4a were not formed.

**Table 1:** Optimization of reaction conditions for synthesis of imine and amide from amines and alcohols^a^

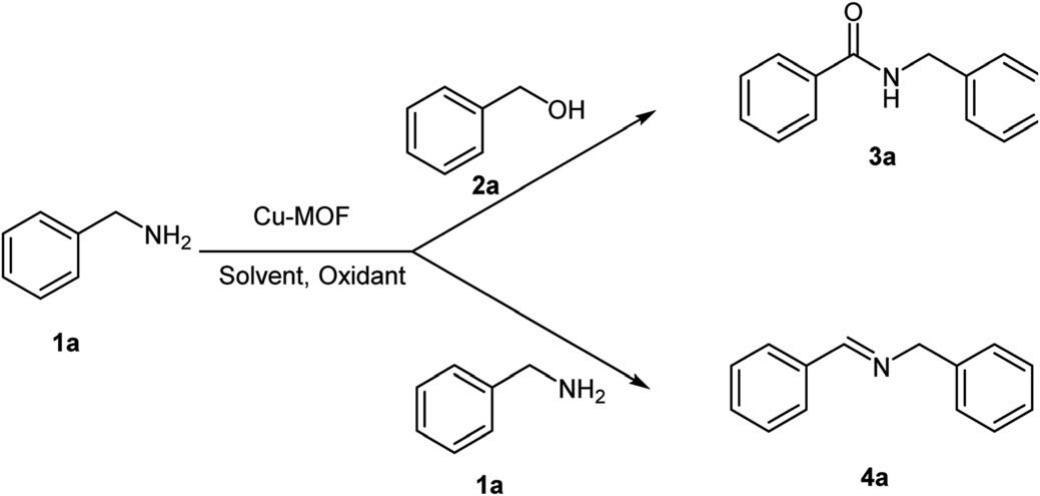					
Entry	Catalyst Cu-MOF (mol%)	Oxidant	Solvent	Yield 3a (%)	Yield 4a (%)
1	5	TBHP	CH_3_CN	52	35
2	10	TBHP	CH_3_CN	60	40
3	20	TBHP	CH_3_CN	65	50
4	5	TBHP	THF	75	60
**5**	**10**	**TBHP**	**THF**	**86**	**79**
6	20	TBHP	THF	81	74
7	5	TBHP	DMF	10	25
8	10	TBHP	DMF	25	35
9	20	TBHP	DMF	25	35
10	—	TBHP	THF	—	—
11	10	—	THF	—	—

a Reaction condition: benzylamine 1a (1.0 mmol), benzyl amine 1a or benzyl alcohol 2a (1.0 mmol), TBHP (2.0 mmol), solvent (5 ml), time: 24 h, rt.

After optimization of the model reaction in hand (Table 1, entry 5), to know the substrate scope a range of benzylic amine and benzylic alcohols for the synthesis of amides (Table 2) were used under the optimized reaction conditions. Benzylic alcohols and amines with different substituent provided the corresponding amides (3a–k) in excellent yields. Propyl amine was favored substrate, provided good yields of amide products.

**Table 2:** Cu-MOF catalyzed dehydrogenative cross-coupling of amines and alcohols to amides^a^

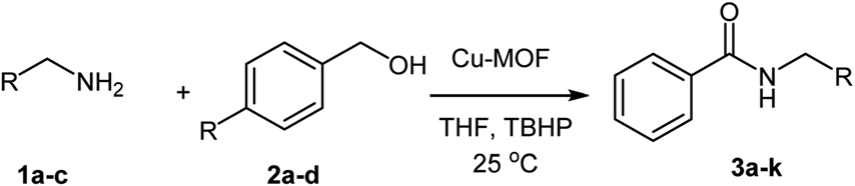				
Entry	Amine	Alcohols	Product 3	Yield (%)
1	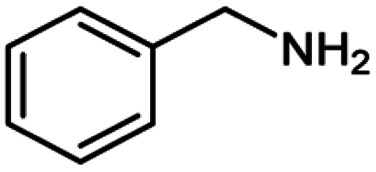	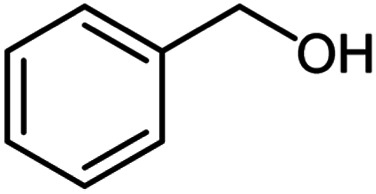	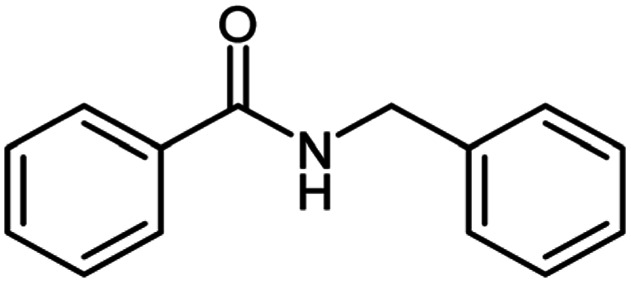	78
2	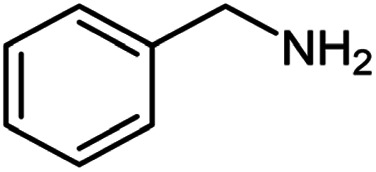	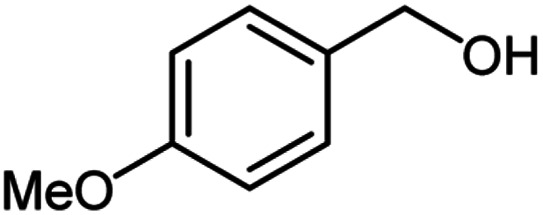	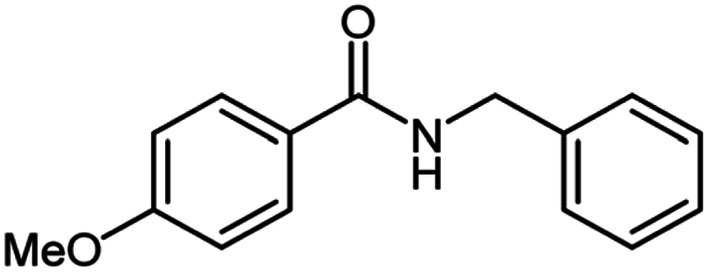	72
3	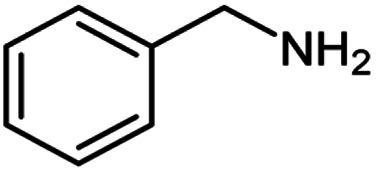	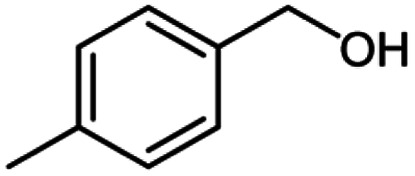	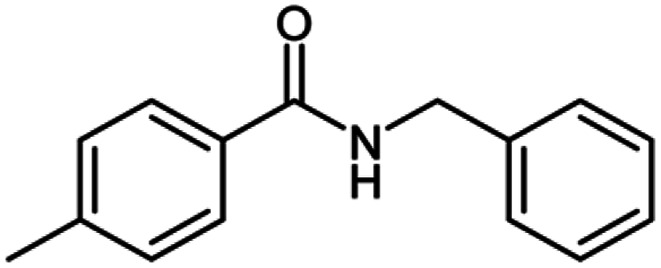	70
4	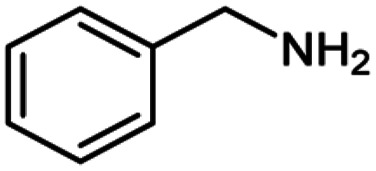	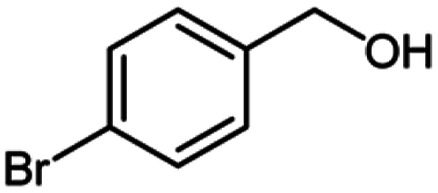	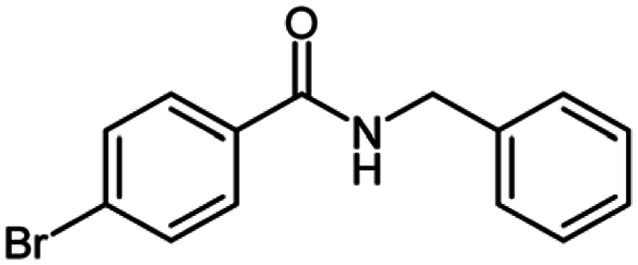	74
5	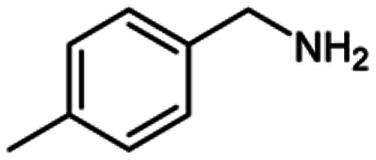	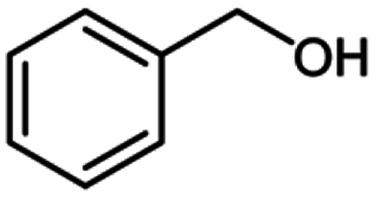	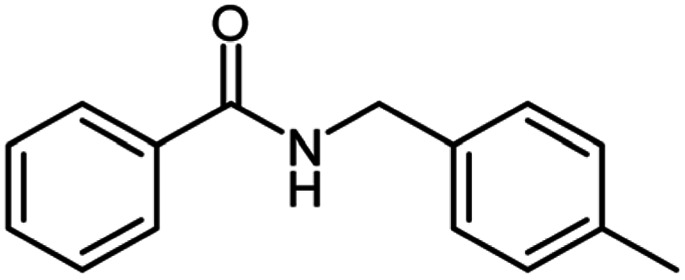	65
6	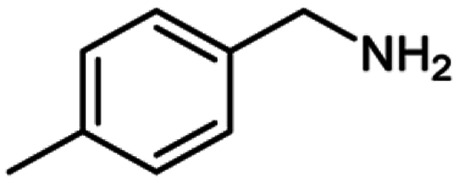	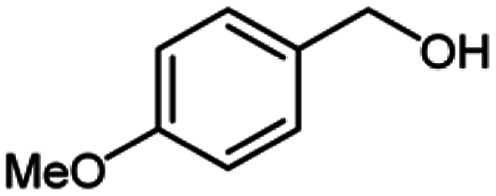	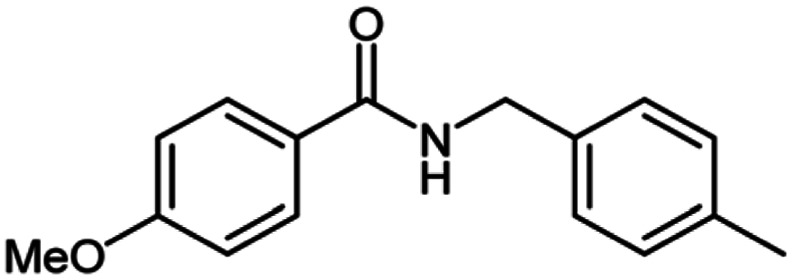	62
7	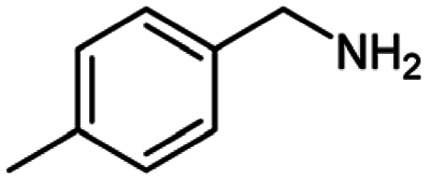	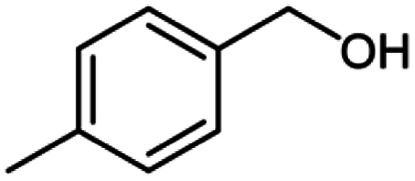	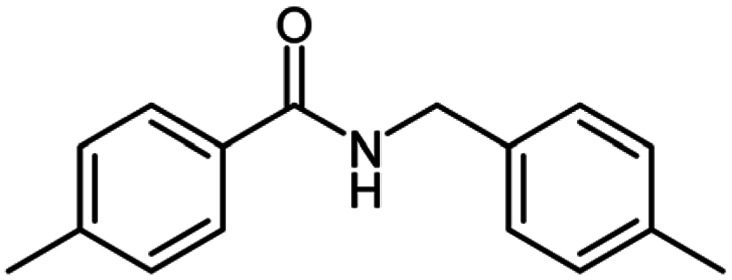	76
8	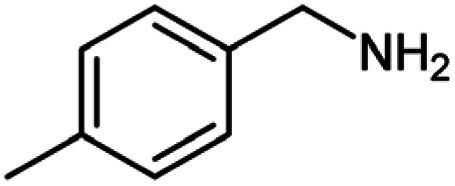	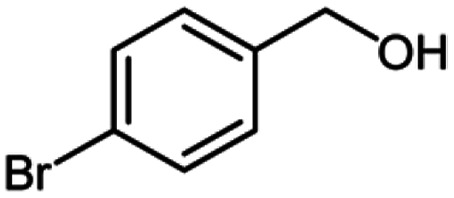	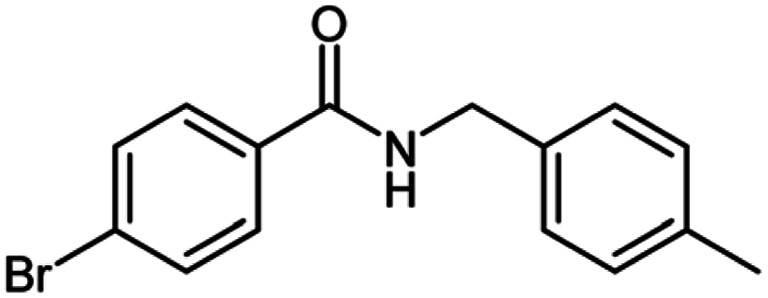	72
9	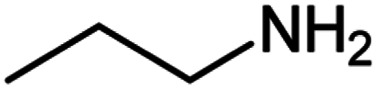	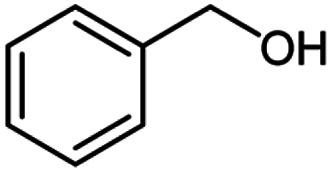	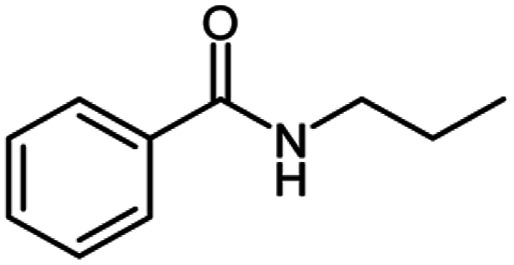	74
10	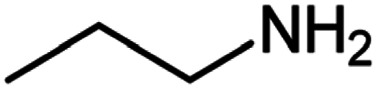	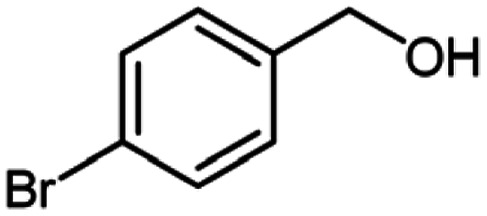	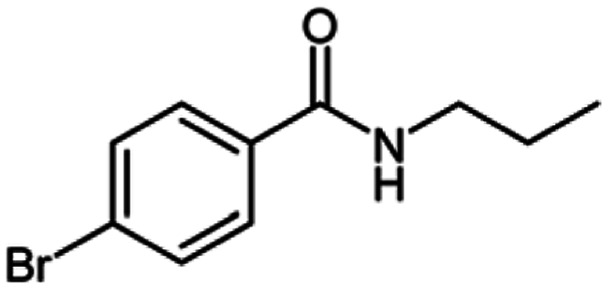	72
11	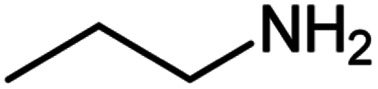	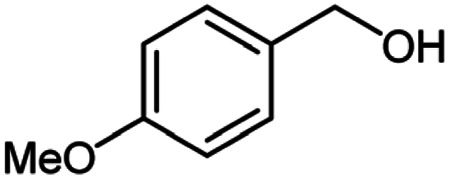	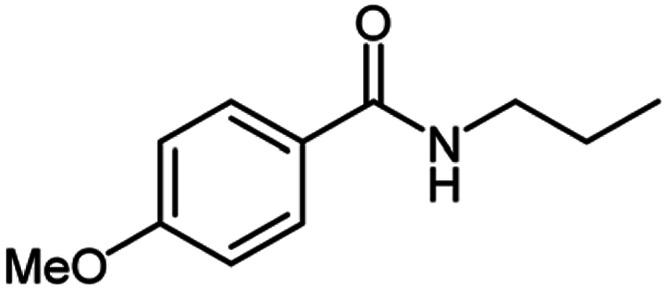	69

a Reaction conditions: 1 (1.0 equiv.), 2 (1.0 equiv.), TBHP (2.0 equiv.), Cu-MOF (10 mol%) in THF (5 ml) at 25 °C for 24 h.

For the investigation of substrate scope of oxidative dehydrogenative homo-coupling of benzyl amines to imines, benzyl amines with electron-donating group at *ortho* and *para* position were used and imines were formed in good yields (Table 3, entries 1–7) but benzyl amines with electron-withdrawing group at *para* position led to formation of a trace of product (Table 3, entry 8).

**Table 3:** Cu-MOF catalyzed dehydrogenative homo-coupling of amines to imines^a^

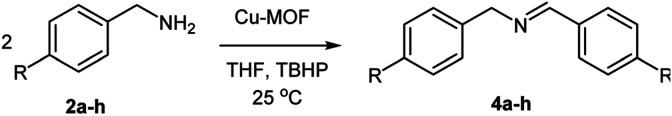			
Entry	Amine 2	Product 4	Yield (%)
1	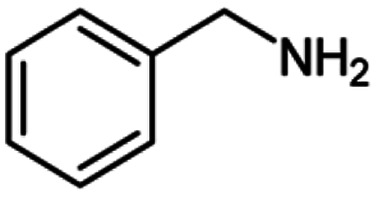	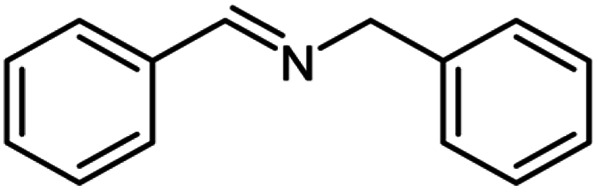	75
2	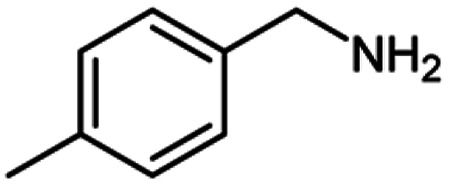	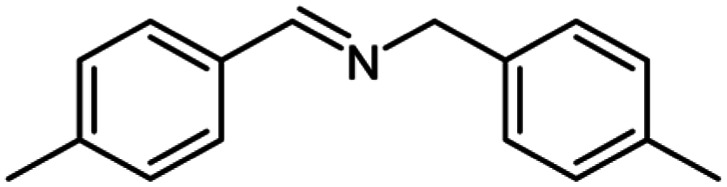	69
3	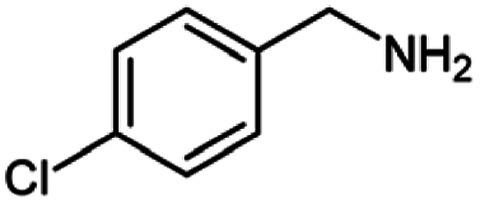	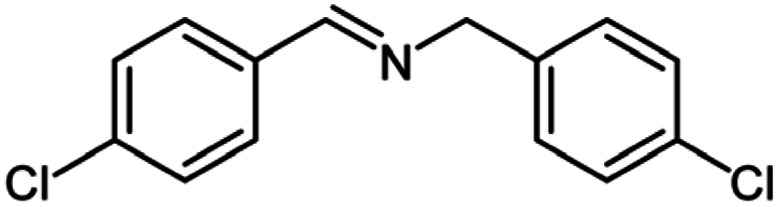	68
4	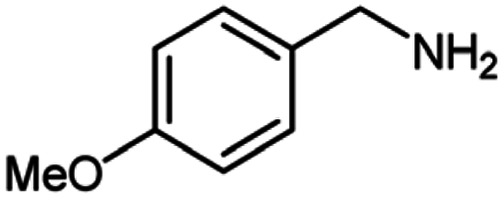	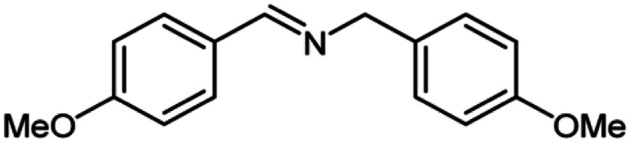	65
5	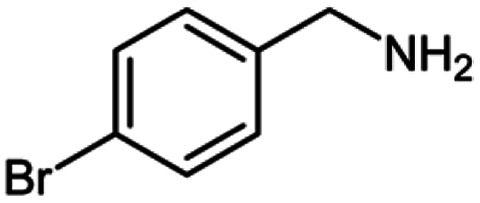	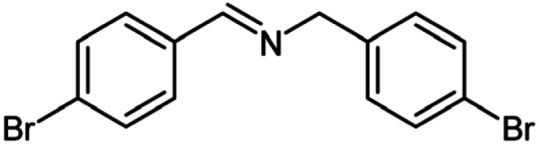	72
6	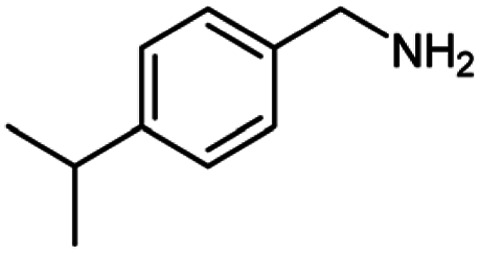	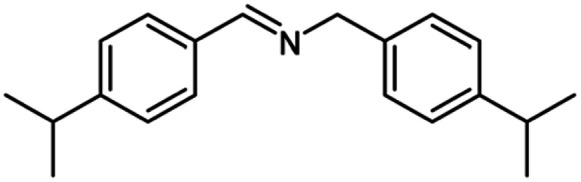	71
7	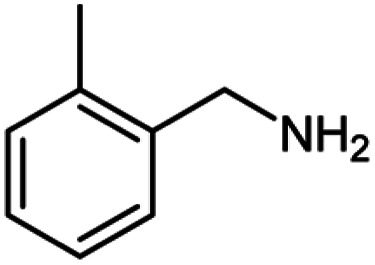	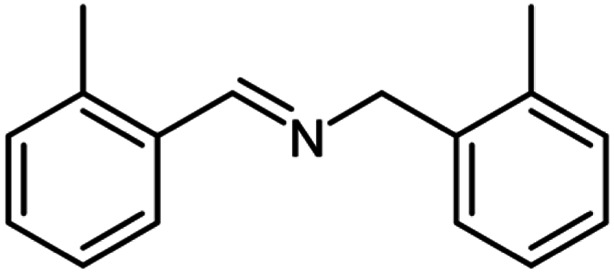	76
8	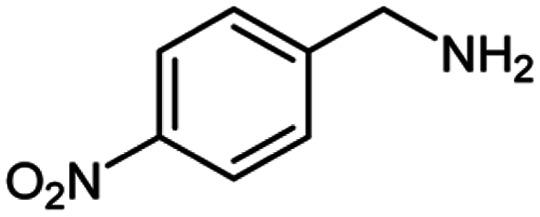	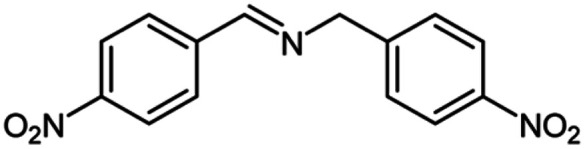	Trace

a Reaction conditions: 2 (1.0 equiv.), TBHP (2.0 equiv.), Cu-MOF (10 mol%) in THF (5 ml) at 25 °C for 24 h.

**Scheme 2:**
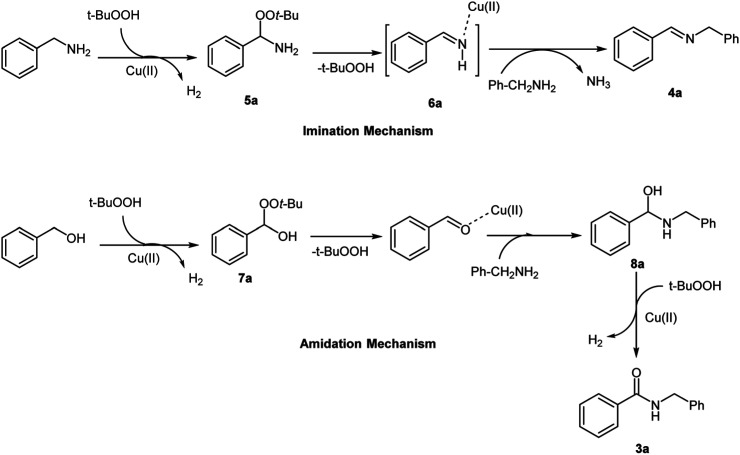
Proposed mechanism for the synthesis of amides and imines catalyzed by Cu-MOF.

A possible mechanism for these type of reactions is shown in Scheme 2. Cu(ii) oxidized TBHP in such a way that TBHP transformed into *tert*-butylproxy radical. *tert*-Butylproxy radical captured a hydrogen from benzylamine or benzyl alcohol, thereby converting the benzylamine and benzyl alcohol into 5a and 7a proxy intermediates. Elimination of TBHP from this two intermediates led to imine 6a and benzaldehyde. Addition of benzyl amine to imine 6a and elimination of ammonia resulted imine 3a. Also, aminal 8a was obtained by addition of benzyl amine to benzaldehyde and continued oxidative dehydrogenation of aminal 8a by Cu(ii) and TBHP led to amide 4a. The latter mechanism was confirmed by reaction between benzaldehyde and benzylamine under the same conditions and led to exclusive formation of the corresponding amide. This indicates that the reaction proceeds through an aldehyde. On the other hand, as shown in Table 1 products 3 and 4 were not formed in the absence of Cu-MOF and TBHP as catalyst and oxidant and all of these evidence supports our proposed mechanism.

The Cu-MOF catalyst also shown good recyclability and stability. The catalyst was recovered by simple filtration and washed with methanol and dried in oven and reused for 4 times (Table 4). The catalyst could be stored for a long time under air atmosphere without significant loss of catalytic activity. Also, the XRD pattern of Cu-MOF shows that the crystalline structure of Cu_2_(BDC)_2_DABCO is maintained after four run (Fig. S1†).^46*a*^

**Table 4:** Recovery and reuse of Cu-MOF in the dehydrogenative coupling of amines and alcohols

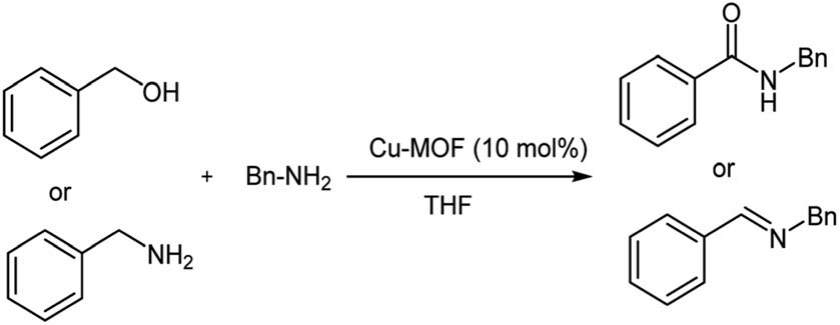				
Run	1	2	3	4
Yield (%)	75	75	74	72

A comparison with other catalytic systems in the dehydrogenative homo- or cross-coupling of amines and alcohols demonstrated that our present Cu-MOF catalyst system exhibited a higher conversion and yield under milder conditions (Table 5).

**Table 5:** Comparison of activity for different catalytic systems in the coupling of benzyl alcohol and benzyl amine

Entry	Catalyst	Reaction condition	Yield (compound 3a or 4a)	Ref. no.
1	Au_6_Pd/resin	NaOH (1.1 eq.), O_2_ balloon, H_2_O, r.t., 2 h	51% (3a)	48
2	Ru(COD)Cl_2_/PCyp_3_.HBF_4_	KO*t*Bu, toluene, 110 °C, 24 h	78% (3a)	49
3	Cu_2_O/CQDs	CH_3_CN, O_2_, white cold LED *λ* > 400 nm, 24 h	95% (4a)	50
4	Co_2_(CO)_8_/trioctylphosphine oxide (TOPO)	Mesitylene, 164 °C, 24 h	79% (4a)	51
5	Silicagel supported salicylic acid	O_2_ (0.1 MPa), toluene, 90 °C, 24 h	81% (4a)	52
6	Manganese pincer complex	KO*t*Bu, toluene, 110 °C, 48 h	88% (3a)	53
7	Cu-MOF	TBHP, THF, r.t	86% (3a), 75% (4a)	Present work

## Conclusion

In conclusion, we have identified Cu-MOF as a green and recyclable heterogeneous catalyst for the efficient dehydrogenative coupling of alcohols with amines for the synthesis of amides and imines. A different range of amines and alcohols are applicable in this two reactions. Furthermore, this cost-effective reaction provides practical alternatives for the synthesis of amides and imines under the mild conditions. Further studies on the catalytic application MOFs are in progress and would be presented in the future.

## Conflicts of interest

There are no conflicts to declare.

## Supplementary Material

RA-011-D1RA03142B-s001
