# Supplementary material for: Direct synthesis of amides and imines by dehydrogenative homo or cross-coupling of amines and alcohols catalyzed by Cu-MOF
Source: RSC Adv. 2021 Jun 10;11(34):20788–93. doi: 10.1039/d1ra03142b (PMC9034032; doi:10.1039/d1ra03142b)
Supplement: RA-011-D1RA03142B-s001 [file RA-011-D1RA03142B-s001.pdf]

Electronic Supplementary Material (ESI) for RSC Advances.
This journal is © The Royal Society of Chemistry 2021

Supporting Information

Direct Synthesis of Amides and Imines by Dehydrogenative Homo or Cross-Coupling of Amines and Alcohols catalyzed by Cu-MOF

Soheil Zamani Anbardan[a], Javad Mokhtari*[a], Ahmad Yari [a], Abolfazl Hasani Bozcheloei [a]

Department of Chemistry, Science and Research Branch. Islamic Azad University, P.O. Box 14515/775, Tehran, Iran. Corresponding author e-mail address: j.mokhtari@srbiau.ac.ir.

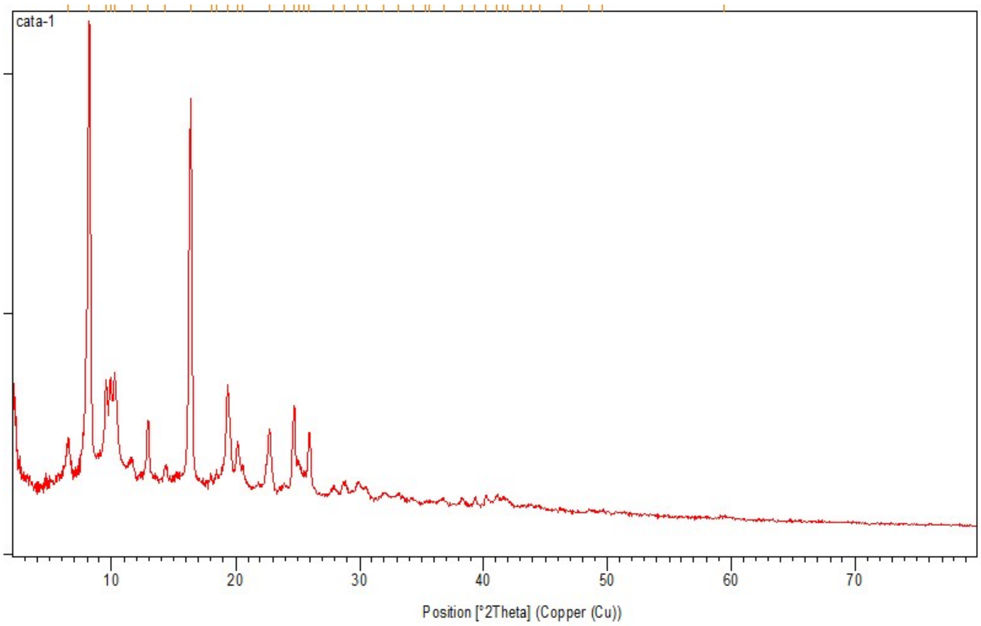

Figure S1. XRD pattern of recycled $Cu_2(BDC)_2DABCO$

***N*-benzylbenzamide**:

White solid; ^{1}H NMR (500 MHz, $CDCl_3$): δ 7.80 (d, J = 7.1 Hz, 1H, CH of Ar), 7.43−7.51 (m, 3H, CH of Ar), 7.36−7.37 (m, 4H, CH of Ar), 7.29 (d, J = 7.1 Hz, 2H), 6.44 (s, 1H, NH), 4.65 (s, 2H, Benzylic CH_2).

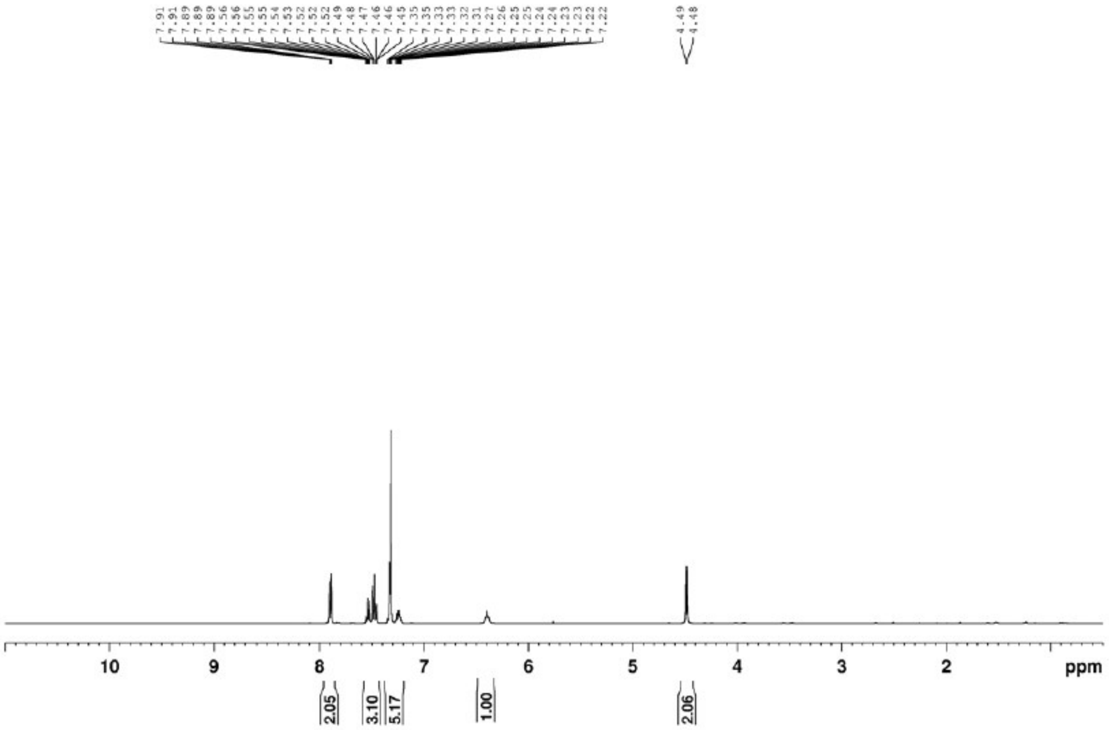

Figure S2. ^{1}H-NMR spectrum of *N*-benzylbenzamide

***N*-benzyl-4-methoxybenzamide:**

White solid; ^{1}H NMR (500 MHz, $CDCl_3$): δ 7.76 (d, J = 8.9 Hz, 2H, CH of Ar), 7.36−7.37 (m, 3H, CH of Ar), 7.28−7.30 (m, 2H, CH of Ar), 6.96 (d, J = 8.9 Hz, 2H), 6.42 (s, 1H, NH), 4.82 (s, 2H, Benzylic CH_2), 3.87 (S, 3H, OMe).

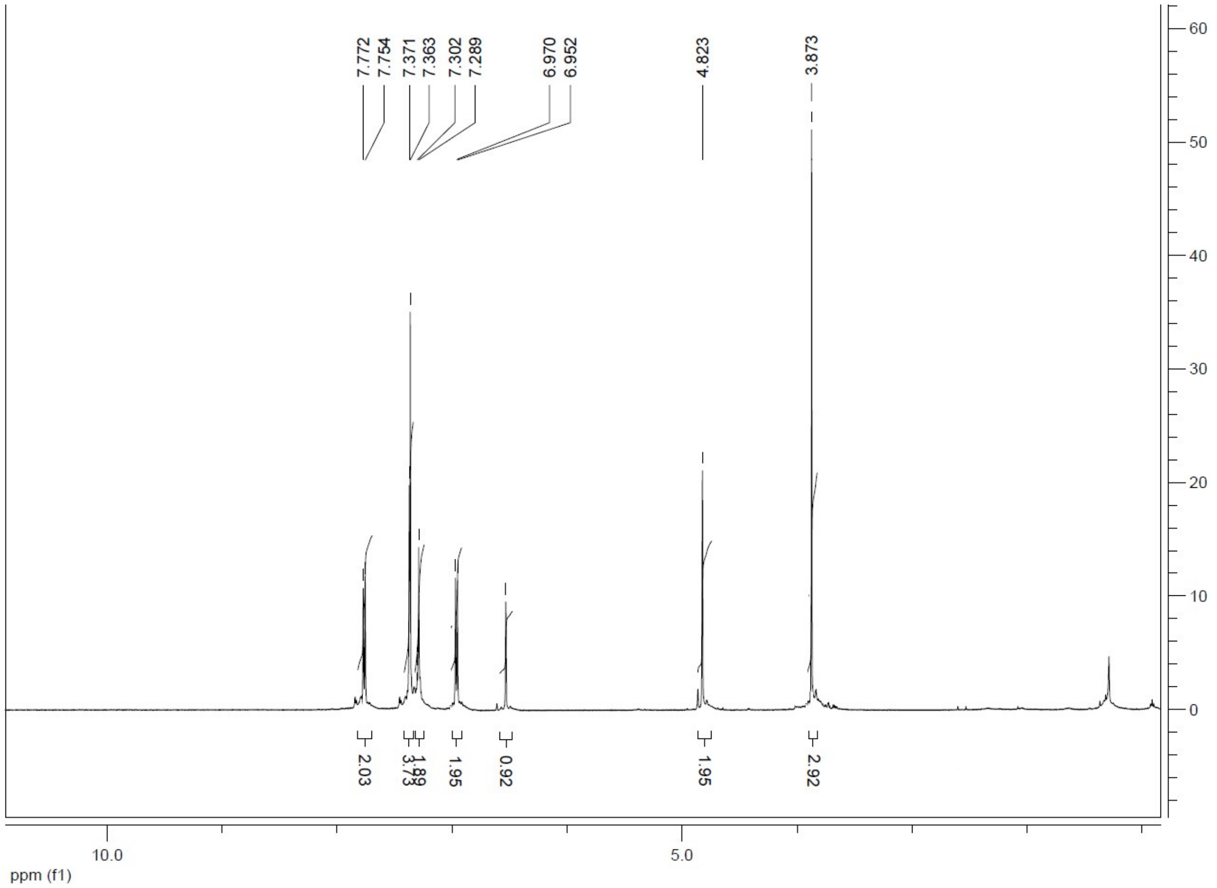

Figure S3. ^{1}H-NMR spectrum of N-benzyl-4-methoxybenzamide

***N*-benzyl-4-methylbenzamide:**

White solid;^{1}H NMR (500 MHz, $CDCl_3$): δ 7.69 (d, ^{3}j= 8.0 Hz, 2H, CH of Ar), 7.27-7.36 (m, 5H, CH of Ar), 7.22 (d, J= 8.0 Hz, 2H, CH of Ar), 6.49 (bs, 1H, NH), 4.63 (d, 2H, J = 5.0 Hz, Benzylic CH_2), 2.39 (s, 3H, Me).

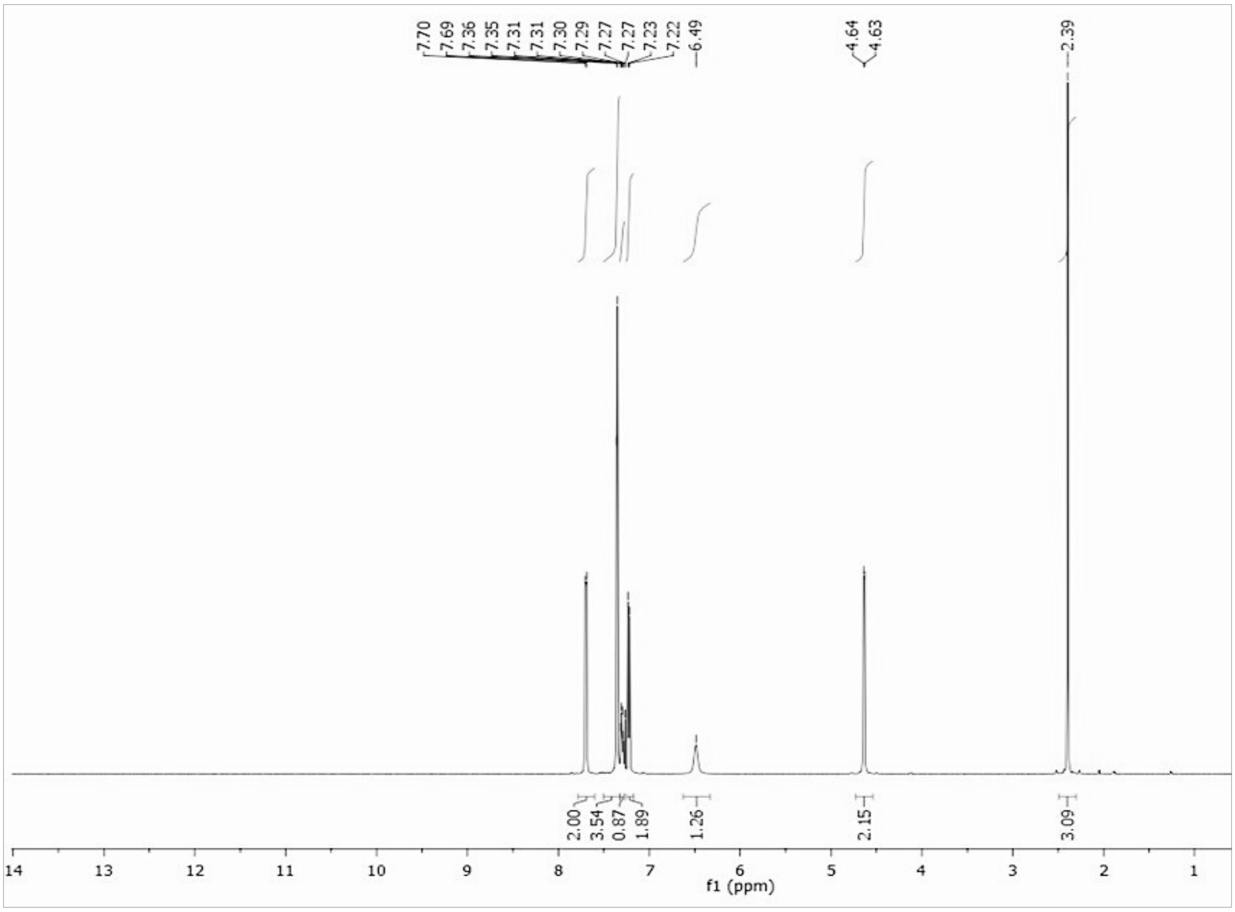

Figure S4. ^{1}H-NMR spectrum of N-benzyl-4-methylbenzamide

***N*-benzyl-4-bromobenzamide:**

Pale yellow solid; ^{1}H NMR (500 MHz, $CDCl_3$): δ 7.66-7.68 (m, 2H, CH of Ar), 7.57-7.59 (m, 2H, CH of Ar), 7.31-7.39 (m, 5H, CH of Ar), 6.42 (bs, 1H), 4.65 (d, 2H, J = 5.5 Hz).

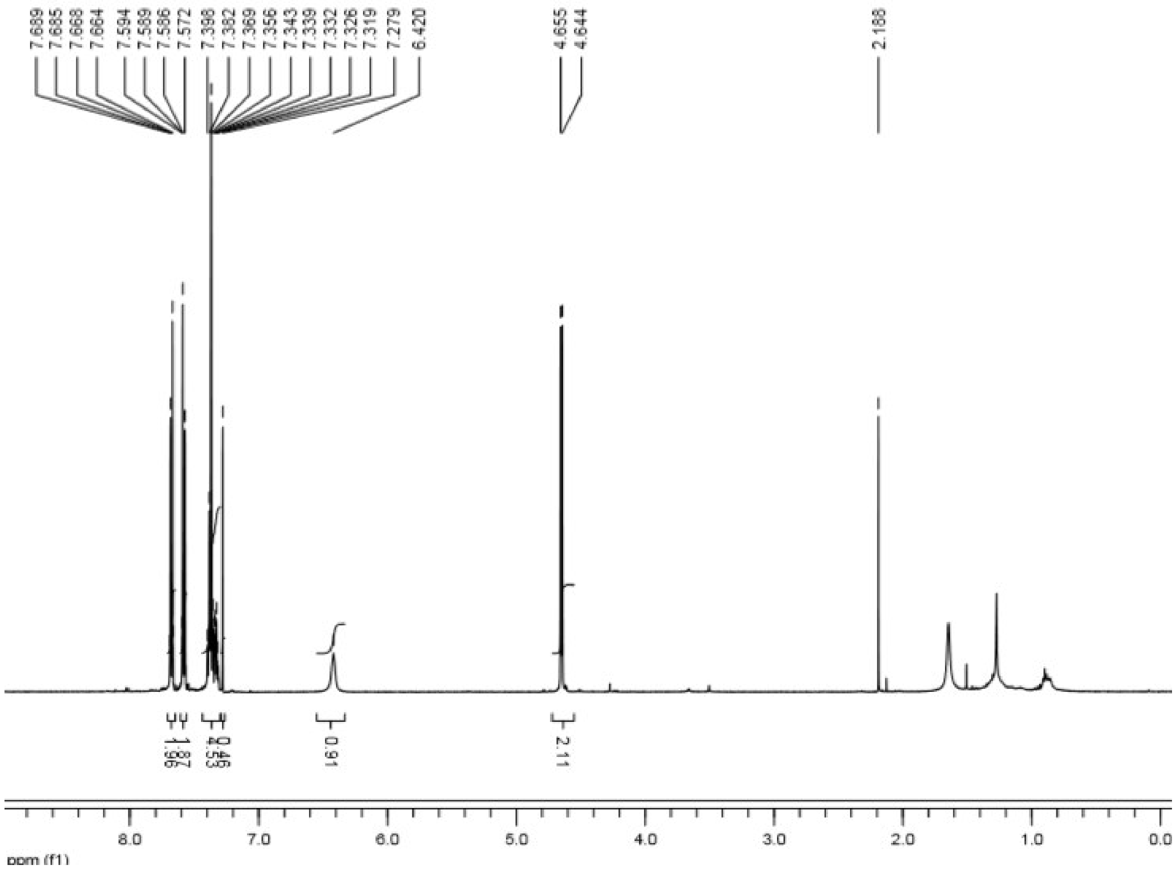

Figure S5. ^{1}H-NMR spectrum of *N*-benzyl-4-bromobenzamide

***N*-(4-methylbenzyl)benzamide**:

White solid; ^{1}H NMR (500 MHz, $CDCl_3$): δ 7.88 (d, J= 7.5 Hz, 2H, CH of Ar), 7.45-7.55 (m, 3H, CH of Ar), 7.12-7.22 (m, 4H, CH of Ar), 6.49 (bs, 1H, NH), 4.43 (d, 2H, J= 4 Hz, Benzylic CH_2), 2.27 (s, 3H, Me).

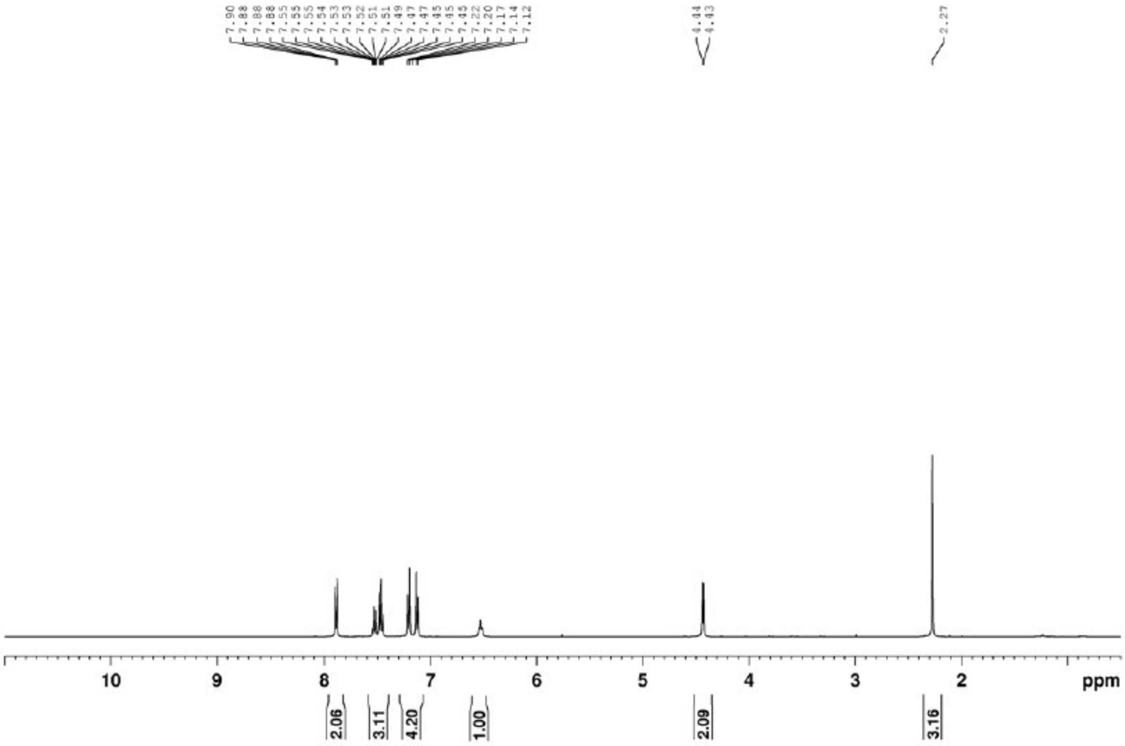

Figure S6. ^{1}H-NMR spectrum of N-(4-methylbenzyl)benzamide

4-methoxy-*N*-(4-methylbenzyl)benzamide:

White solid; ^{1}H NMR (500 MHz, $CDCl_3$): δ 7.43-7.44 (m, 3H, CH of Ar), 7.36-7.37 (m, 3H, CH of Ar), 7.27-7.29 (m, 3H, CH of Ar), 6.42 (s, 1H, NH), 4.85 (s, 2H, Benzylic CH_2), 3.81 (s, 3H, OMe), 2.39 (s, 3H, Me).

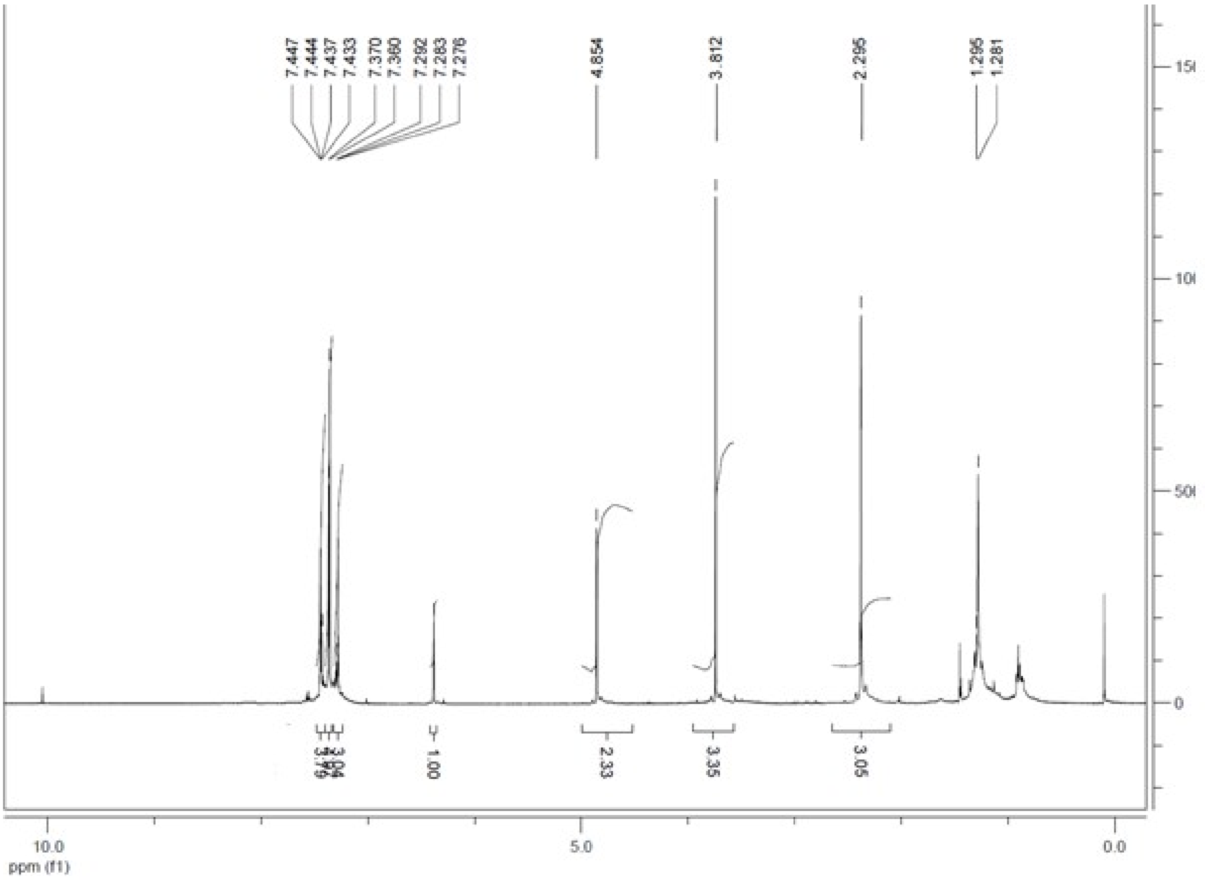

Figure S7. ^{1}H-NMR spectrum of 4-methoxy-N-(4-methylbenzyl)benzamide

4-methoxy-*N*-(4-methylbenzyl)benzamide:

White solid; ^{1}H NMR (500 MHz, $CDCl_3$): δ 7.81-7.82 (m, 1H, CH of Ar), 7.43-7.45 (m, 3H, CH of Ar), 7.37-7.38 (m, 3H, CH of Ar), 7.28-7.31 (m, 1H, CH of Ar), 6.43 (s, 1H, NH), 4.86 (s, 2H, Benzylic CH_2), 2.63 (s, 3H, Me), 2.32 (s, 3H, Me).

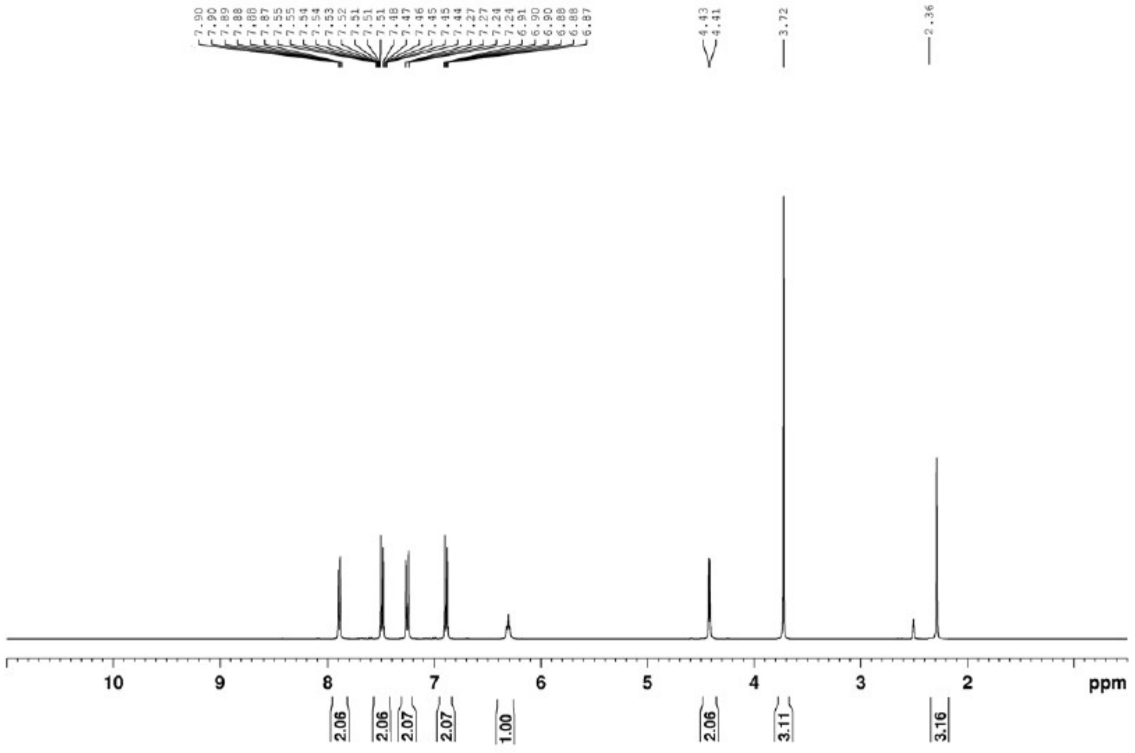

Figure S8. ^{1}H-NMR spectrum of *N*-4-methylbenzyl4-methylbenzamide

***N*-propylbenzamide:**

White solid; ^{1}H NMR (500 MHz, $CDCl_3$): δ 7.36-7.37 (m, 3H, CH of Ar), 7.28-7.30 (m, 2H, CH of Ar), δ 6.32 (s, 1H, NH), 3.43 (t, 2H, J = 7.4 Hz, CH_2), 1.72 (q, 2H, J = 7.4 Hz, CH_2), δ 0.94 (t, 3H, J = 7.4 Hz, Me).

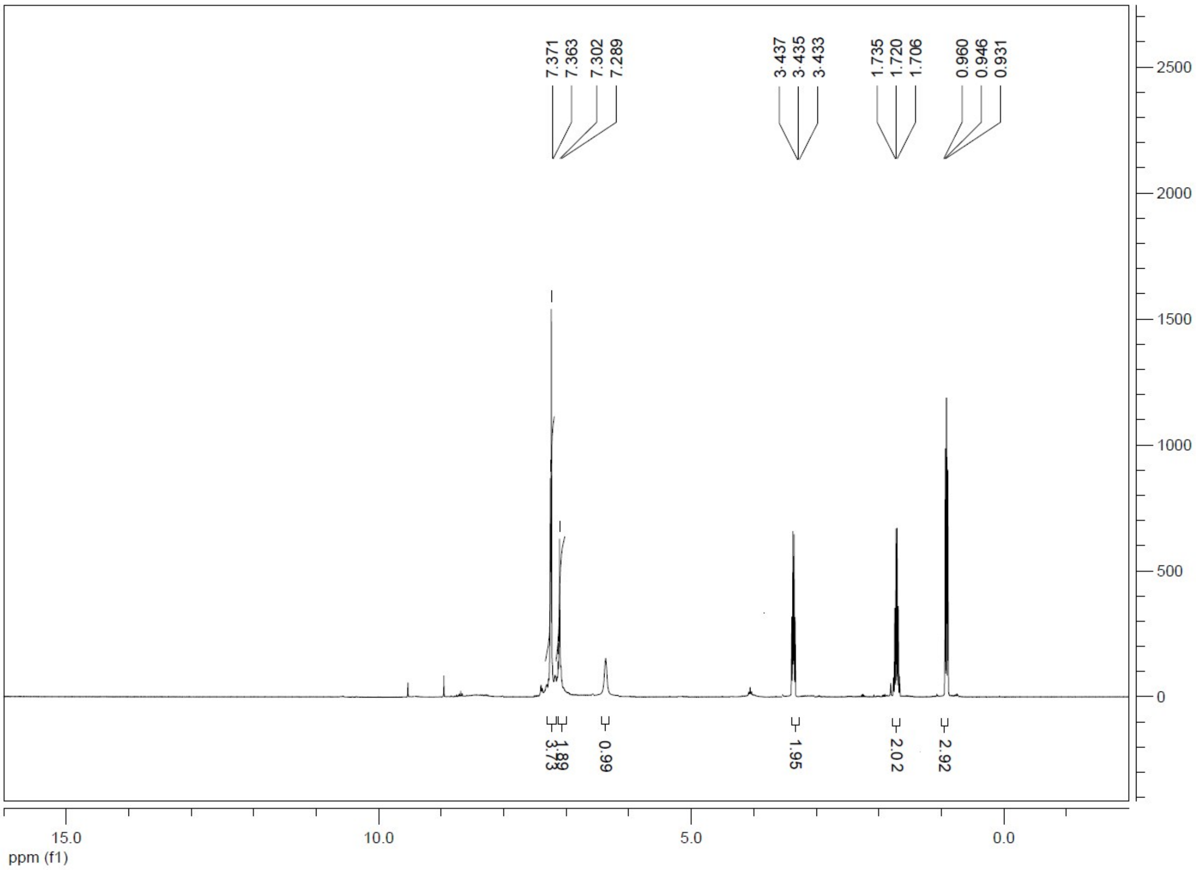

Figure S9. ^{1}H-NMR spectrum of N-propylbenzamide

4-bromo-*N*-propylbenzamide:

White solid; ^{1}H NMR (500 MHz, $CDCl_3$): δ 7.72-7.73 (d, 2H, J = 8.5 Hz, CH of Ar), 7.430-7.434(d, 2H, J = 8.5 Hz, CH of Ar), 6.10 (s, 1H, NH), 3.43-3.44 (m, 2H, CH_2), 1.70-1.73 (m, 2H, CH_2), 0.93-0.96 (m, 3H, Me).

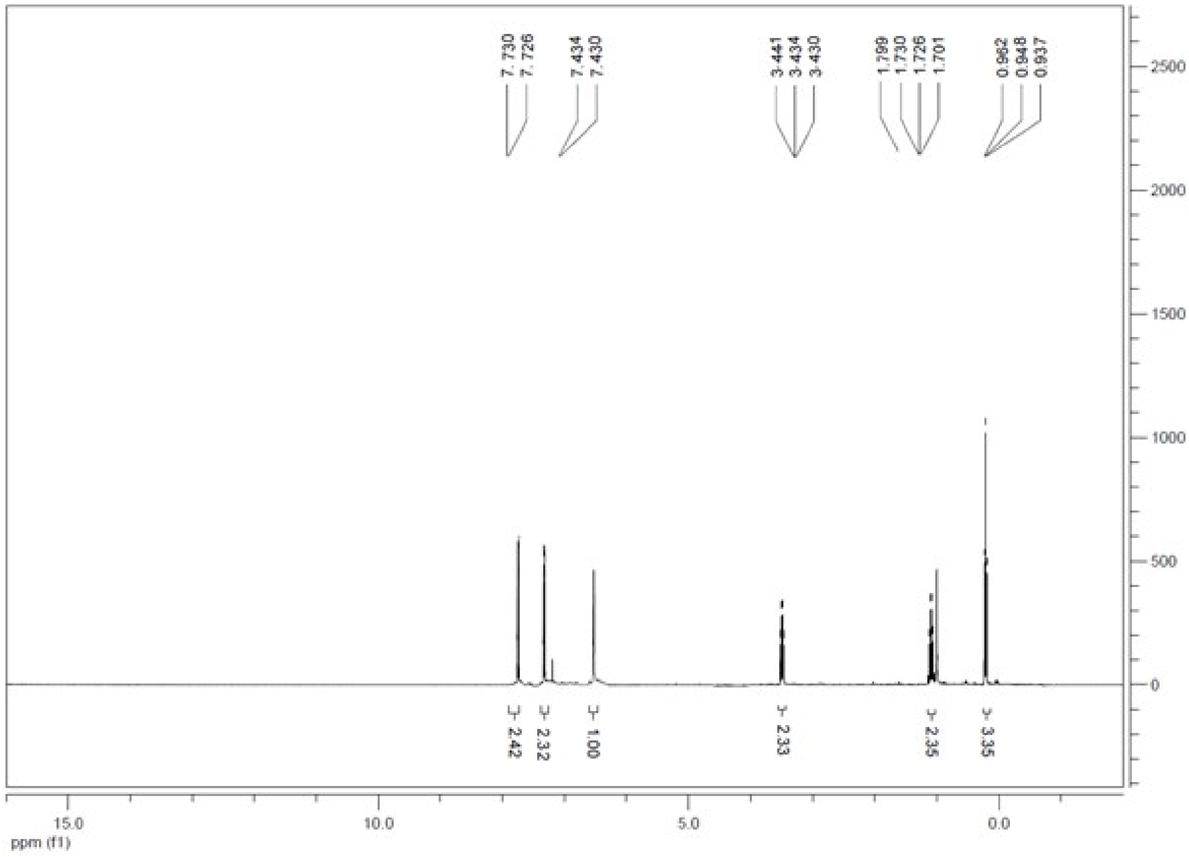

Figure S10. ^{1}H-NMR spectrum of 4-bromo-N-propylbenzamide

4-methoxy-*N*-propylbenzamide:

White solid; ^{1}H NMR (400 MHz, $CDCl_3$): δ 7.72 (d, 2H, J = 8.0 Hz, CH of Ar), 6.87 (d, 2H, J = 8.0 Hz, CH of Ar), 6.31 (s, 1H, NH), 3.81 (s, 3H, OMe), 3.37-3.41 (m, 2H, CH_2), 1.40-1.56 (m, 2H, CH_2), 0.90-0.94 (m, 3H, Me).

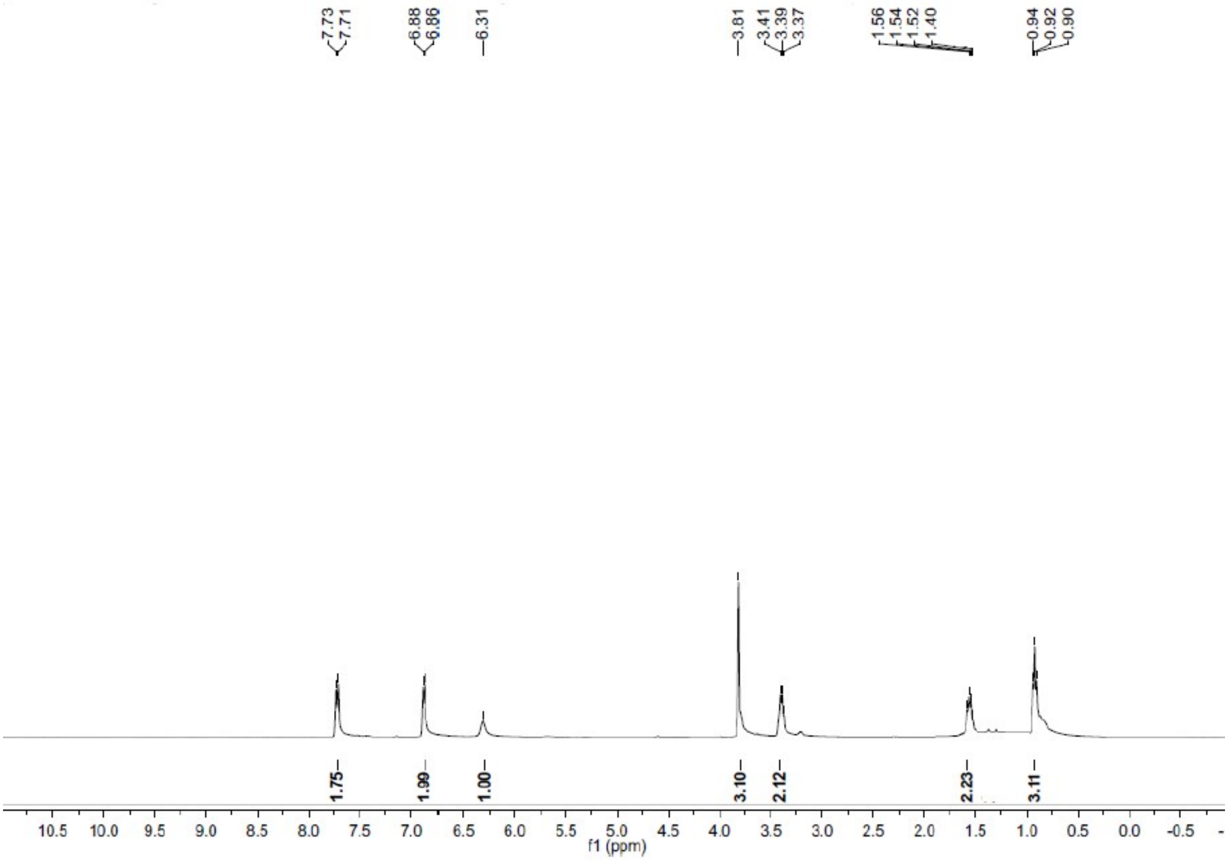

Figure S11. ^{1}H-NMR spectrum of 4-methoxy-*N*-propylbenzamide

***N*-Benzylidenebenzylamine**:

^{1}H NMR (400 MHz, CDCl3): δ 8.44 (s, 1H, CH), 7.97 – 7.76 (m, 2H, CH of Ar), 7.57 – 7.42 (m, 3H, CH of Ar), 7.40 (d, J = 4.4 Hz, 4H, CH of Ar), 7.34 – 7.27 (m, 1H, CH of Ar), 4.88 (s, 2H, Benzylic CH_2).

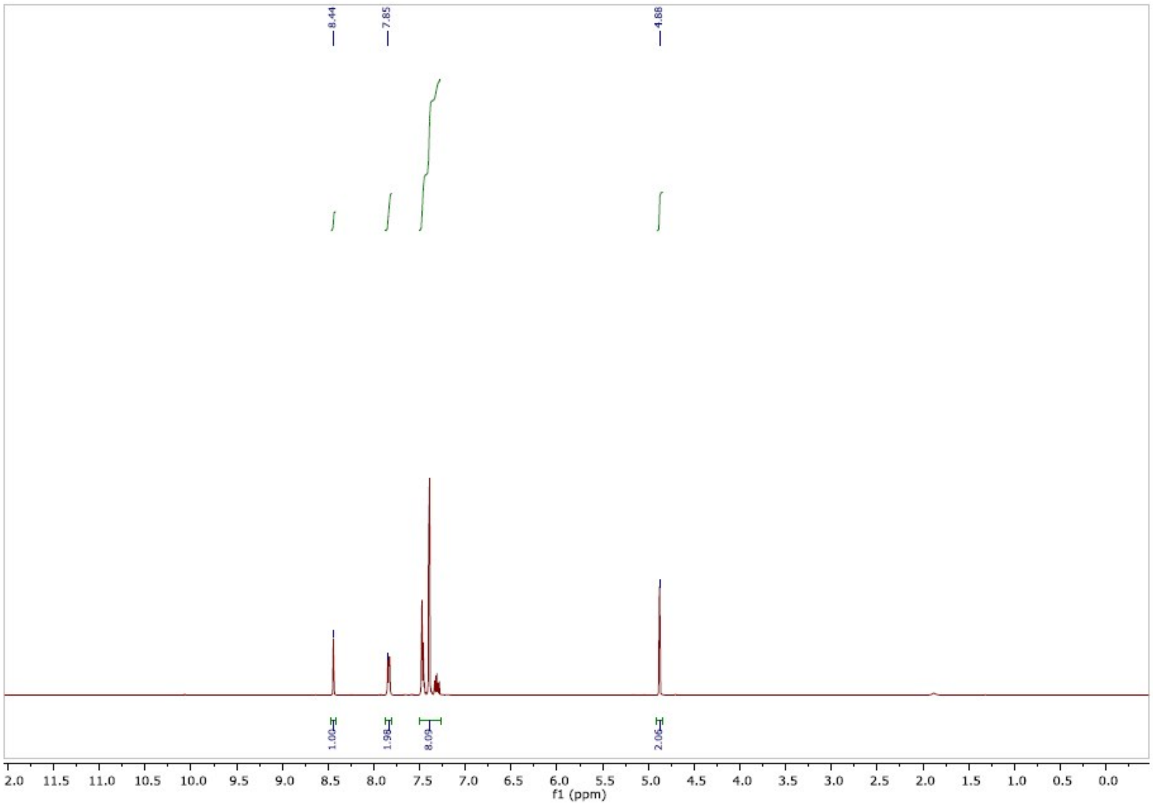

Figure S12. ^{1}H-NMR spectrum of N-Benzylidenebenzylamine

***N*-(4-Methylbenzylidene)-4-methylbenzylamine:**

^{1}H NMR (400 MHz, $CDCl_3$): δ ppm: 8.36 (s, 1H, CH), 7.69 (d, J = 8.1 Hz, 2H, CH of Ar), 7.28 – 7.21 (m, 4H, CH of Ar), 7.20 – 7.14 (m, 2H, CH of Ar), 4.79 (s, 2H, Benzylic CH_2), 2.40 (s, 3H, Me), 2.36 (s, 3H, Me).

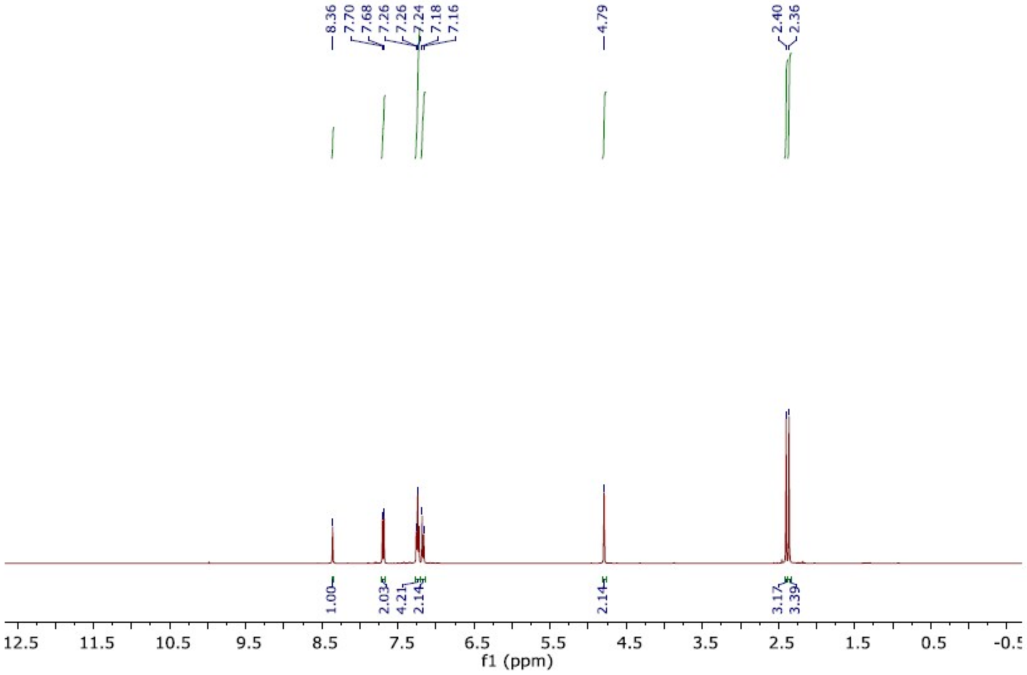

Figure S13. ^{1}H-NMR spectrum of *N*-(4-Methylbenzylidene)-4-methylbenzylamine

N-(4-Chlorobenzylidene)-4-chlorobenzylamine:

1H NMR (400 MHz, $CDCl_3$): δ ppm: 8.34 (s, 1H, CH), 7.74 – 7.68 (m, 2H, CH of Ar), 7.42 – 7.37 (m, 2H, CH of Ar), 7.35–7.24 (m, 4H, CH of Ar), 4.77 (s, 2H, Benzylic CH2).

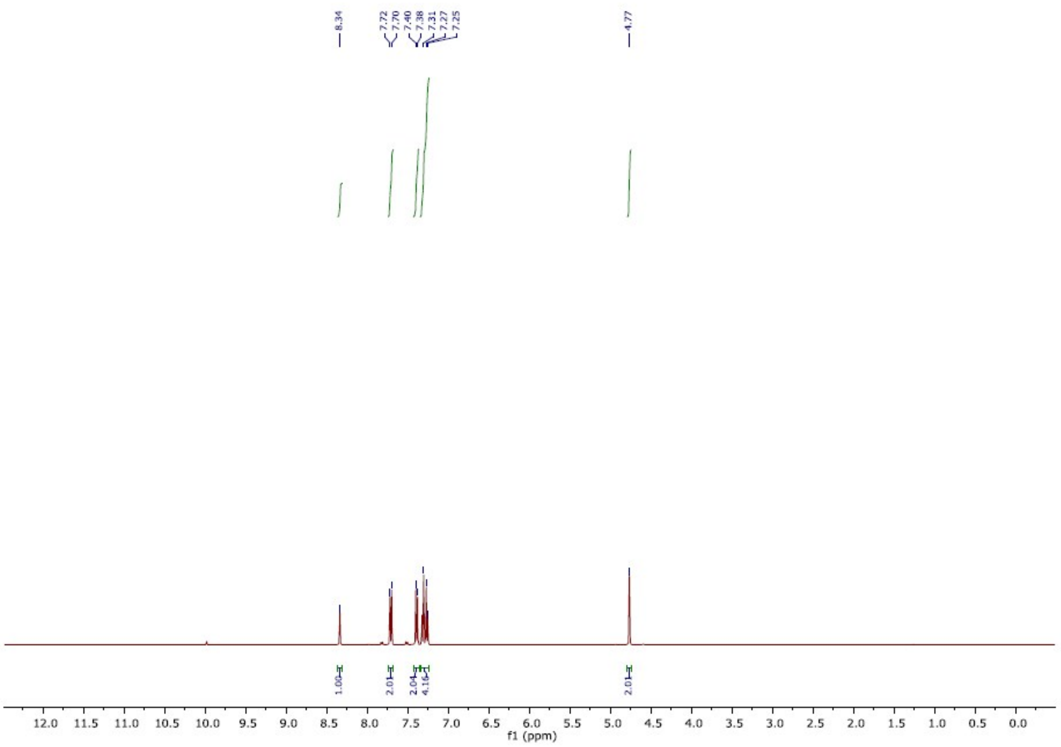

Figure S14. ^{1}H-NMR spectrum of N-(4-Chlorobenzylidene)-4-chlorobenzylamine

N-(4-methoxybenzylidene)-4-methoxybenzylamine:

^{1}H NMR (400 MHz, $CDCl_3$): δ ppm: 8.33 (s, 1H, CH), 7.63 – 7.65 (m, 2H, CH of Ar), 7.54 – 7.57 (m, 2H, CH of Ar), 7.46–7.48 (m, 2H, CH of Ar), 7.20–7.22 (m, 2H, CH of Ar), 4.75 (s, 2H, Benzylic CH2), 3.86 (s, OMe), 3.84 (s, OMe).

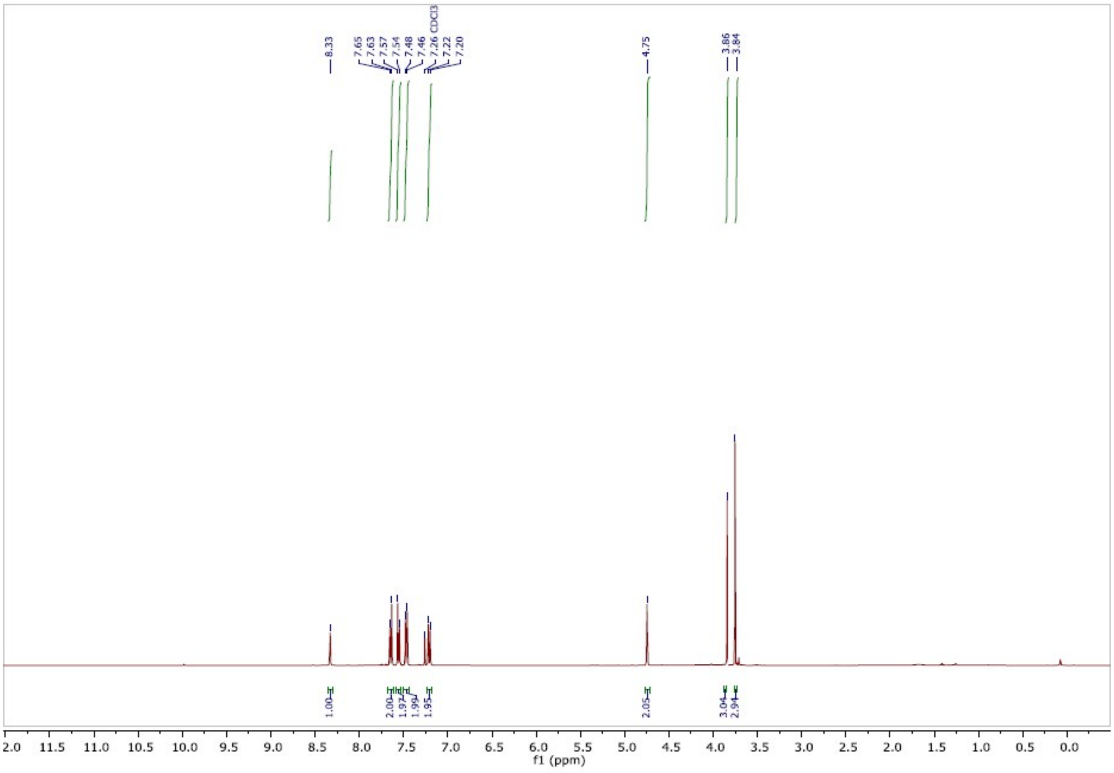

Figure S15. ^{1}H-NMR spectrum of N-(4-methoxybenzylidene)-4-methoxybenzylamine

N-(4-bromobenzyl)-1-(4-bromophenyl)methanimine

^{1}H NMR (400 MHz, $CDCl_3$) δ ppm: 8.35 (s, 1H, CH), 7.74-7.81 (m, 2H, CH of Ar), 7.27–7.33 (m, 2H, CH of Ar), 7.00 – 7.14 (m, 4H, CH of Ar), 4.77 (s, 2H, Benzylic CH_2).

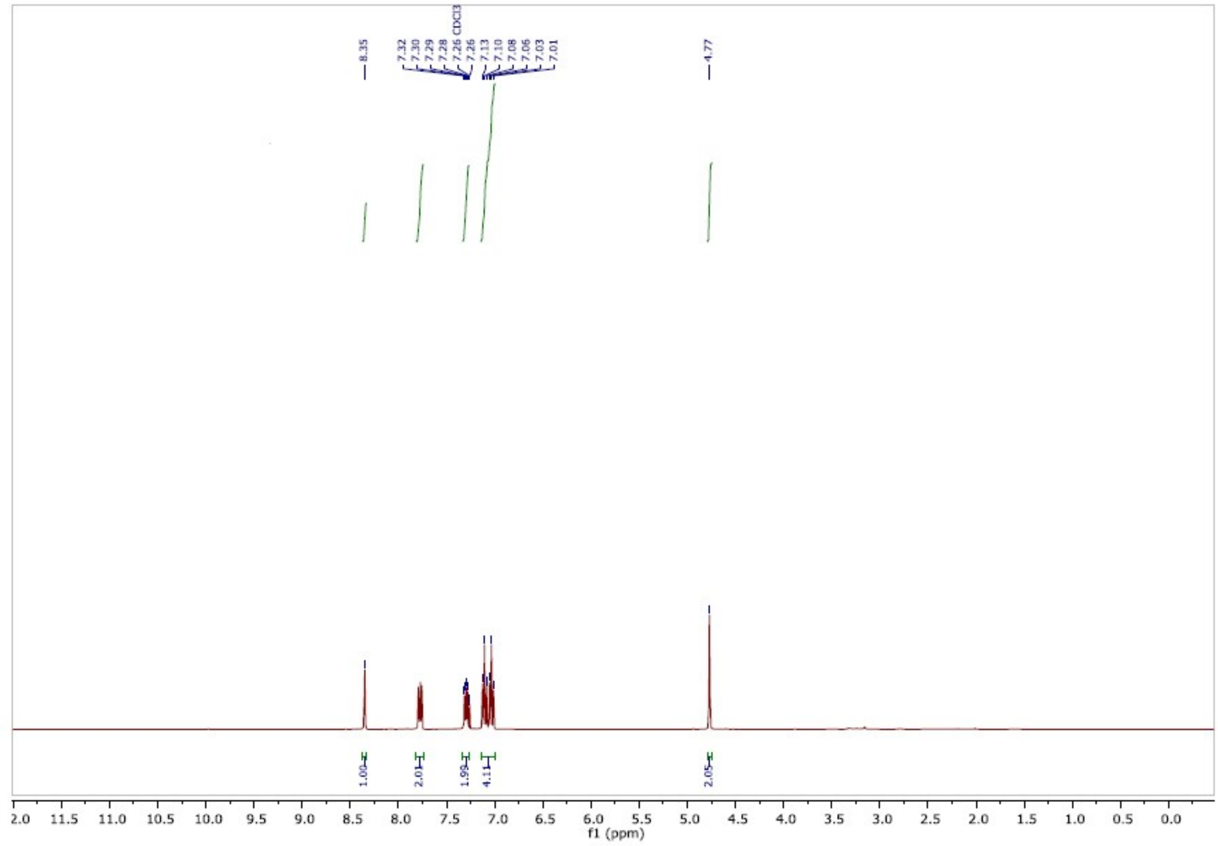

Figure S16. ^{1}H-NMR spectrum of N-(4-bromobenzyl)-1-(4-bromophenyl)methanimine

***N*-(2-methylbenzyl)-1-(o-tolyl)methanimine**

^{1}H NMR (400 MHz, $CDCl_3$) δ ppm: 8.45 (s, 1H, CH), 7.77-7.85 (m, 2H, CH of Ar), 7.22-7.30 (m, 4H, CH of Ar), 6.90-6.96 (m, 2H, CH of Ar), 4.75 (s, 2H, Benzylic CH_2), 2.42 (s, 3H, CH_3), 2.29 (s, 3H, CH_3).

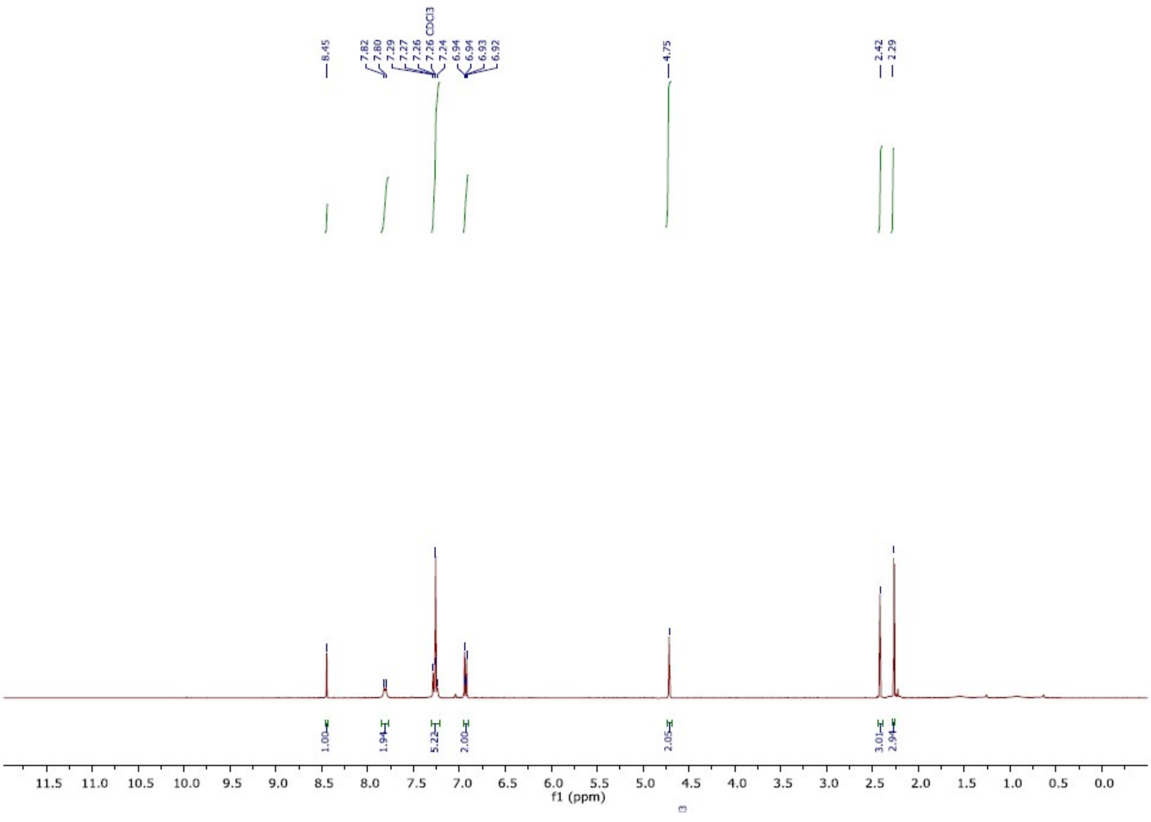

Figure S17. ^{1}H-NMR spectrum of N-(2-methylbenzyl)-1-(o-tolyl)methanimine

N-(4-isopropylbenzyl)-1-(4-isopropylphenyl)methanimine:

^{1}H-NMR (400 MHz, $CDCl_3$) δ ppm: 8.32 (s, 1H, CH), 7.65-7.89 (m, 2H, CH of Ar), 7.28-7.35 (m, 2H, CH of Ar), 7.23-7.25 (m, 2H, CH of Ar), 7.06-7.23 (m, 2H, CH of Ar), 5.47 (s, 2H, Benzylic CH_2), 3.55-3.61 (m, 2H, CH), 1.25 (d, 6H, ^3J=6.5 Hz, CH_3), 1.29 (d, 6H, ^3J=6.5 Hz, CH_3).

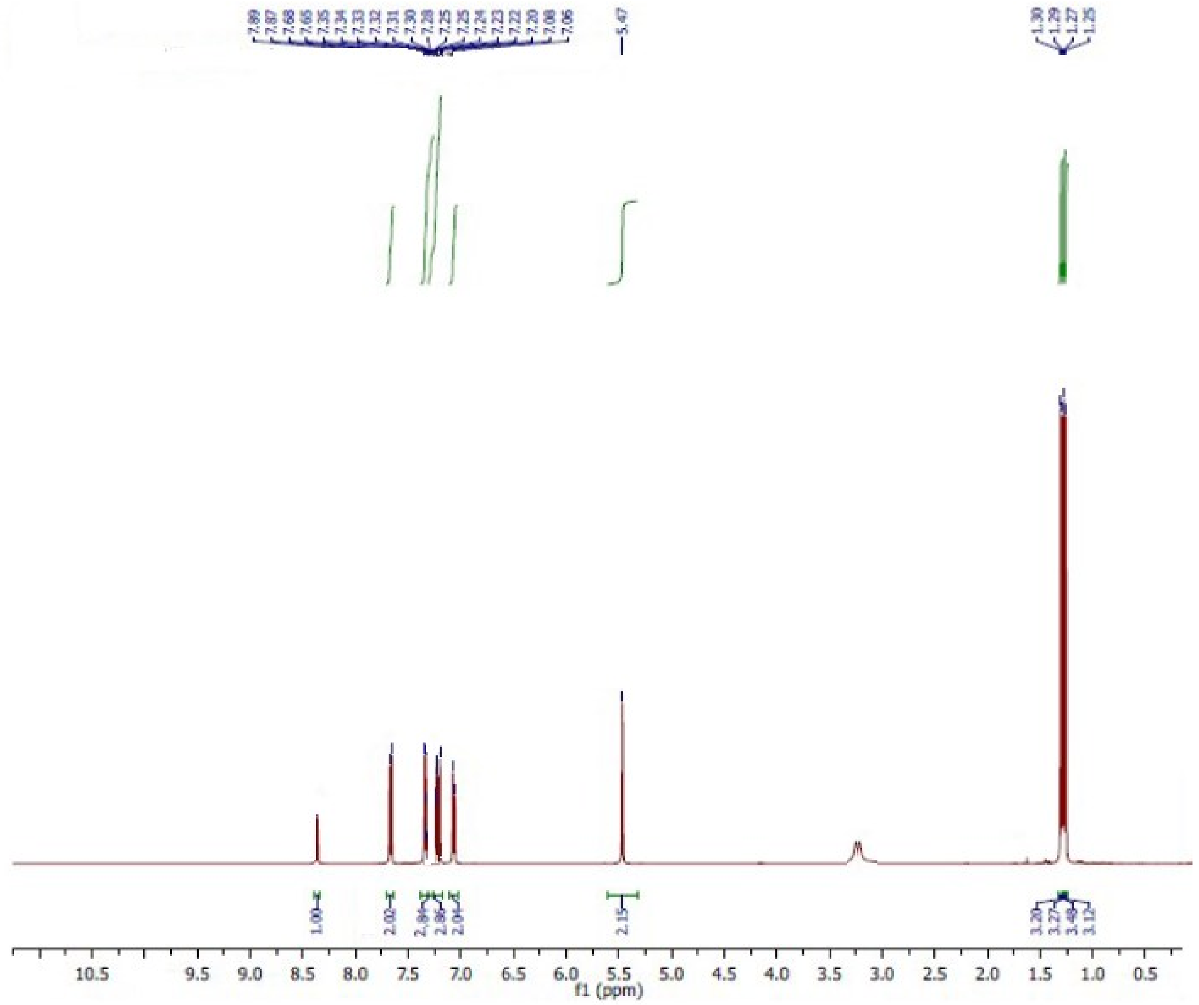

Figure S18. N-(4-isopropylbenzyl)-1-(4-isopropylphenyl)methanimine
